# Adherence to oral endocrine therapy in racial/ethnic minority patients with low socioeconomic status before and during the COVID-19 pandemic

**DOI:** 10.1007/s11096-023-01609-6

**Published:** 2023-06-28

**Authors:** Sama Rahimi, Onyebuchi Ononogbu, Anjana Mohan, Daniel Moussa, Susan Abughosh, Meghana V. Trivedi

**Affiliations:** 1 Department of Pharmacy Practice and Translational Research, University of Houston, College of Pharmacy, Health 2, 4349 Martin Luther King Blvd, Houston, TX 77204-5000 USA; 2 Department of Pharmaceutical Outcomes and Policy, University of Houston College of Pharmacy, Houston, TX USA

**Keywords:** Aromatase inhibitor, Breast cancer, COVID-19 pandemic, Medication adherence, Racial and ethnic minority, Tamoxifen

## Abstract

**Background:**

Adherence to oral endocrine therapy (OET) is crucial in ensuring its maximum benefit in the prevention and treatment of hormone receptor-positive (HR +) breast cancer (BC). Medication use behavior is suboptimal especially in racial/ethnic minorities with lower socioeconomic status (SES).

**Aim:**

We aimed to assess the impact of the coronavirus disease 2019 (COVID-19) pandemic on OET adherence and identify demographic and/or clinical characteristics associated with nonadherence in racial/ethnic minorities with lower SES.

**Method:**

A retrospective study was conducted at the Harris Health System in Houston, Texas. Data were collected during the 6 months before and 6 months after the start of the pandemic. The adherence was assessed using the prescription refill data using the proportion of days covered. A multivariable logistic regression model was used to identify demographic/clinical characteristics associated with nonadherence. Eighteen years or older patients on appropriate doses of OET for prevention or treatment of BC were included.

**Results:**

In 258 patients, adherence was significantly lower during the pandemic (44%) compared to before the pandemic (57%). The demographic/clinical characteristics associated with OET nonadherence before the pandemic were Black/African American, obesity/extreme obesity, prevention setting, tamoxifen therapy, and 4 or more years on OET. During the pandemic, prevention setting and those not using home delivery were more likely to be nonadherent.

**Conclusion:**

OET adherence was significantly reduced during the COVID-19 pandemic in racial/ethnic minority patients with low SES. Patient-centered interventions are necessary to improve OET adherence in these patients.

## Impact statements

- The adherence to OET for breast cancer prevention or treatment was reduced in racial/ethnic minority patients with low socioeconomic status during the COVID-19 pandemic.
- Patients taking OET for prevention have worse adherence compared to those taking it for treatment of HR+ BC; Black/African Americans had worse adherence than Hispanic patients.
- Patients who used home delivery had better adherence during the pandemic, suggesting that this intervention may be useful even during the non-pandemic times.

## Introduction

Hormone receptor-positive (HR +) breast cancer (BC) is the most common subtype found in about 80% of BC patients [1–3]. In HR + BC patients, better adherence to oral endocrine therapy (OET) results in superior outcomes [4–9]. Despite a 30–50% reduction in recurrence risk by 5–10 years of OET, nonadherence rate is high among the patients [10–12]. The Food and Drug Administration (FDA)-approved OETs used are tamoxifen and Aromatase Inhibitors (AIs) such as anastrozole, exemestane, and letrozole [13–18]. OETs are also used in the prevention setting for patients considered at higher risk of BC and have shown to reduce the risk of new BC diagnoses by ~ 40% [19]. Nonadherence to OET is more common among racial/ethnic minority groups compared to non-Hispanic whites [20–25] and in patients with financial burdens [21, 23, 26]. Higher OET nonadherence, reported in up to 80% of racial/ethnic minorities and low-income patients, likely contributes to the disparity in their outcomes [27–29].

Cancer health disparity has been a significant societal burden in the United States (U.S.) and is further magnified during the recent coronavirus disease 2019 (COVID-19) pandemic. Before the pandemic, high out-of-pocket medical costs and high unemployment rates due to cancer were common causes of treatment refusal or nonadherence in cancer patients [30, 31]. The COVID-19 pandemic has further increased the stress level of already vulnerable cancer patients because of the fear of contracting the virus and inadequate access to and utilization of health care, especially in those with low SES [32–34]. Therefore, accessible and equitable cancer care has been markedly compromised in racial/ethnic minority cancer patients with low SES during the pandemic [35, 36]. The impact of the COVID-19 pandemic and barriers to OET adherence in racial/ethnic minority patients with low SES are poorly understood despite the established clinical benefit of OET adherence.

### Aim

The goal of this study was to assess the impact of the COVID-19 pandemic on OET adherence and identify patients and/or tumor characteristics associated with nonadherence in racial/ethnic minorities with lower SES.

### Ethics approval

The study was approved by the institutional review board of the University of Houston (STUDY00001818, approved on Jan 30, 2020) and Harris Health System (Protocol 20-02-2286, approved on March 3, 2020) with a waiver of informed consent.

## Method

### Study design and data source

This retrospective, single-center study included HR + BC patients on OET at Harris Health System in Houston, Texas, which is a county hospital system serving minority patients with low SES. A list of patients of Harris Health System with at least one OET dispensed record from June 2019 through September 2020 was extracted from the EPIC Electronic Health Record (EHR) system. The dispense history from June 2019 through August 2019 was used to determine whether the patients had OET supply in and after September 2019.

### Data management

We collected the following data: patient demographics, height and weight to calculate body mass index (BMI), chronic diseases and comorbidities, diagnoses date, pathological and clinical cancer stage at diagnosis, HR and HER2 status, OET name and dosing, date of first OET prescription and initiation date, OET frequency dispense data, OET dispense quantity, date of OET discontinuation, any change in OET therapy, reason for discontinuation or change, number of month’s supply that was covered using mail-order drug delivery (to patients’ homes) after March 31^st^, 2020, and number of telemedicine appointments after March 31st, 2020. The patients’ prescription refill data included the combination of the prescription dispensed data of any outpatient pharmacy with an integrated e-prescribing system. Thirty patients were included in piloted data collection to review any necessary updates in the data collection form and crosscheck the consistency of data collection by two investigators. Queries were resolved by licensed and board-certified oncology pharmacists (MVT and OO). Random audits were performed during and after data collection to ensure data integrity.

### Study population

Inclusion criteria included patients with at least 18 years of age, seen and followed at Harris Health System, of any stage of HR + BC, and those whose OET was not discontinued due to death or progression. Patients not taking appropriate doses for OET (tamoxifen 20 mg once daily, anastrozole 1 mg once daily, letrozole 2.5 mg once daily, and exemestane 25 mg once daily) or on OET for reasons other than prevention or treatment of HR + BC were excluded.

### Study variables

The primary endpoint was OET adherence during the 6 months prior to (September 2019 through February 2020) and 6 months after the start of the COVID-19 pandemic (April 2020 through September 2020). Adherence to chronic medications including OET could be assessed by calculating the medication possession ratio (MPR) and proportion of days covered (PDC) [37–39]. PDC is a method that avoids the limitation of overestimating adherence for patients who refill their prescriptions early with MPR. PDC was calculated by dividing the number of days patients had OET on hand by the 180 days follow-up period. Patients were considered adherent if they had 80% or more days covered for any OET during the follow-up period [40]. For example, if a patient had filled OET medication for 144 days or more out of the 180 days (follow-up days), then they were considered adherent. Independent variable selection was guided by the Andersen Behavioral Model for Healthcare Resource Use Behavior including predisposing, enabling, and need factors [41]. Predisposing factors included age, race/ethnicity, BMI, type of OET, and years on OET. Enabling factors included home delivery (via mail and free-of-charge to patients) during COVID-19 and the use of telehealth during COVID-19. Need factors included cancer stage, HER2, and comorbidities including metabolic syndrome and depression.

### Statistical analysis

Adherence before and during the pandemic was compared using Student’s paired t-test or McNemar chi-square test when PDC was used as a continuous or categorical variable, respectively. Two multinomial regression models were conducted to identify the demographic/clinical characteristics associated with nonadherence to OET during each of the 6 months periods. The demographic/clinical characteristics included in the model were age, race/ethnicity, BMI, cancer stage, HER2, type of OET, years on OET, metabolic syndrome, and depression. Home delivery during COVID-19 and use of telehealth during COVID-19 were the additional characteristics included in the model during the pandemic. All the statistical analysis was done using Statistical Analysis System (SAS) version 9.4 (SAS Institute, Cary, NC) at a priori significance level of 0.05. The coefficients and resultant odds ratios (odds of an event in the adherent group divided by the odds of an event in the nonadherent group) were automatically generated by the function PROC LOGISTIC in SAS.

## Results

Out of 270 patients, 12 patients were excluded for taking OET for reasons other than HR + BC. The study cohort included 258 patients.

### Baseline characteristics

The baseline demographic and clinical characteristics of patients are presented in Table 1. These characteristics were representative of the patient population of Harris Health System. Among 258 patients that were included in the data analysis, 54 patients were diagnosed with non-invasive (stage 0) BC or ductal/lobular hyperplasia. Out of these 54 patients, 39 (72%) patients had ductal carcinoma in situ (DCIS), 6 (11%) patients had lobular carcinoma in situ (LCIS), 8 (15%) patients had ductal hyperplasia, 1 (2%) had lobular hyperplasia.

**Table 1:** Baseline characteristics (N = 258)

Variable	N (%)
*Age*	
Below 65 years	196 (76.0)
65 years and above	62 (24.0)
*Race/Ethnicity*	
Black/African American	66 (25.6)
Hispanic/Latino	159 (61.6)
White/Caucasian	14 (5.4)
Others	19 (7.4)
*Body Mass Index*	
< 30	98 (38.0)
≥ 30	160 (62.0)
*Stage*	
0 or Ductal/lobular hyperplasia	55 (21.3)
I	71 (27.5)
II	65 (25.2)
III	48 (18.6)
IV	19 (7.4)
*HER2*	
HER2-negative	214 (83.0)
HER2-positive	29 (11.2)
N/A	15 (5.8)
*Oral endocrine therapy*	
Tamoxifen	100 (38.8)
Aromatase Inhibitors	157 (60.8)
N/A	1 (0.4)
*Years on oral endocrine therapy*	
≤ 1 year	120 (46.5)
2–3 years	80 (31.0)
≥ 4 years	58 (22.5)
*Metabolic syndrome*	
No	72 (27.9)
Yes	186 (72.1)
*Depression*	
No	204 (79.1)
Yes	54 (20.9)
*Home Delivery (during COVID-19 pandemic)*	
0	87 (33.7)
≥ 1 home delivery	171 (66.3)
*Telemedicine (during COVID-19 pandemic)*	
0	76 (29.5)
≥ 1	182 (70.5)

### Impact of COVID-19 pandemic on OET adherence

The mean PDC from September 2019 to February 2020 was 0.72 and from April 2020 to September 2020 was 0.52. Of the 258 patients, 112 (43%) patients were nonadherent before the start of COVID-19 pandemic and 171 (66%) patients were nonadherent during the pandemic. There was a significant difference in the adherence before and during the pandemic when PDC was used as a continuous (*p* < 0.0001, Student’s paired t-test) or a categorical variable (*p* < 0.0001, McNemar chi-square test).

### Demographic and clinical characteristics associated with nonadherence before the pandemic

**Fig. 1:**
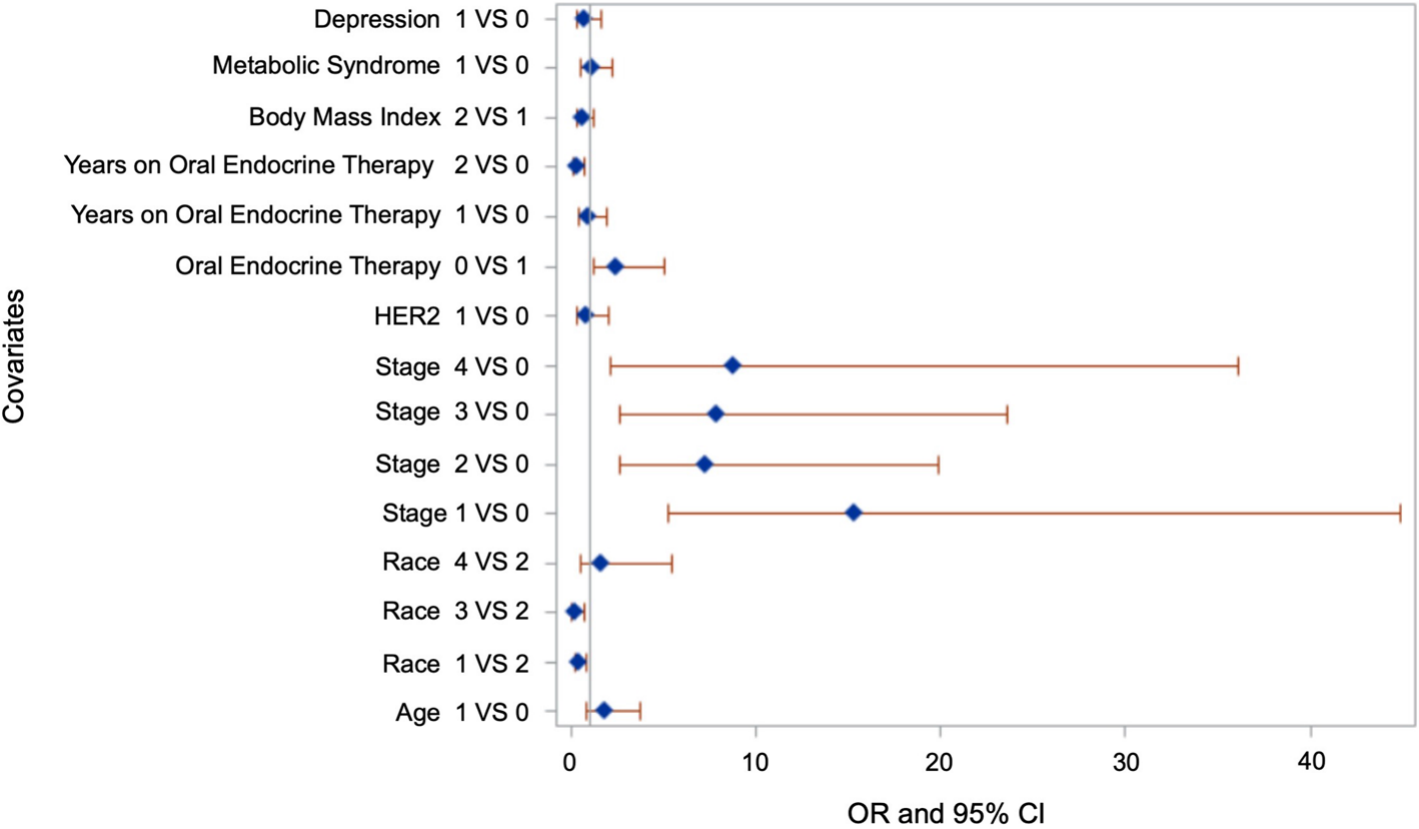
Forest plot for predictors of OET adherence before COVID-19 pandemic. Forest plots were constructed using SAS. The codes for various covariates are as follows: Depression (0 = No, 1 = Yes), Metabolic syndrome (0 = No, 1 = Yes), Body mass index (0 =  < 30 Ref, 1 =  ≥ 30), Years on Oral Endocrine Therapy (0 =  ≤ 1 year, 1 = 2–3 years, 2 =  ≥ 4 years), Oral endocrine therapy (0 = Tamoxifen, 1 = Aromatase inhibitor), HER2 (0 = HER2-negative, 1 = HER2-positive), Stage (0 = stage 0 or Ductal/lobular hyperplasia, 1 = stage I, 2 = stage II, 3 = stage III, 4 = stage IV), Race (1 = Black/African American, 2 = Hispanic/Latino, 3 = White/Caucasian, 4 = Others), Age (0 = Below 65 years, 1 = 65 years and above)

An exploratory multivariable logistic regression analysis of data 6 months before the pandemic identified several factors significantly associated with OET non-adherence. Black/African American and White/Caucasian were less likely to be adherent compared to Hispanic/Latino (Black/African American: odds ratio (OR), 0.43; 95% confidence interval [CI], 0.22–0.84; White/Caucasian: OR, 0.20; 95% CI 0.05–0.73). Obese or extremely obese patients (BMI of ≥ 30) were less likely to be adherent compared to those with a BMI of < 30 (OR, 0.55; 95% CI 0.30–1.01). Patients diagnosed with invasive BC (stages 1–4) were more likely to be adherent compared to those diagnosed with non-invasive tumors or ductal/lobular hyperplasia. Patients on aromatase inhibitors were more likely to be adherent compared to patients on tamoxifen (OR, 2.60; 95% CI 1.26–5.36). Patients taking OET for four years or longer were less likely to be adherent compared to those who were on OET for less than four years (OR, 0.29; 95% CI 0.13–0.65) (Table 2). The forest plot showing the odds-ratio before COVID-19 pandemic is presented as Fig. 1.

**Table 2:** Demographic and clinical characteristics associated with nonadherence to OET before the COVID-19 pandemic

Variable	Adjusted OR	95% CI	*P*-value
*Age*			
Below 65 years	Ref		
65 years and above	1.90	0.91–3.96	0.08
*Race/Ethnicity*			
Hispanic/Latino	Ref		
Black/African American	0.43	0.22–0.84	0.01^a^
White/Caucasian	0.20	0.05–0.73	0.01^a^
Others	1.53	0.45–5.11	0.48
*Body Mass Index*			
< 30	Ref		
≥ 30	0.55	0.30–1.01	0.05^a^
*Stage*			
0 or Ductal/lobular hyperplasia	Ref		
I	13.34	4.71–37.73	< 0.0001^a^
II	6.74	2.52–17.99	0.0001^a^
III	7.45	2.55–21.74	0.0002^a^
IV	7.95	1.96–32.25	0.003^a^
*HER2*			
HER2-negative	Ref		
HER2-positive	0.85	0.33–2.16	0.73
*Oral endocrine therapy*			
Tamoxifen	Ref		
Aromatase Inhibitors	2.60	1.26–5.36	0.009^a^
*Years on oral endocrine therapy*			
≤ 1 year	Ref		
2–3 years	0.78	0.40–1.51	0.46
≥ 4 years	0.29	0.13–0.65	0.002^a^
*Metabolic syndrome*			
No	Ref		
Yes	1.11	0.56–2.18	0.75
*Depression*			
No	Ref		
Yes	0.76	0.36–1.58	0.46

Abbreviations: CI- Confidence interval; OET = Oral endocrine therapy; OR = Odds ratio

A* p*-value ≤ 0.05 was considered statistically significant

^a^Statistically significant

### Demographic and clinical characteristics associated with nonadherence during the pandemic

**Fig. 2:**
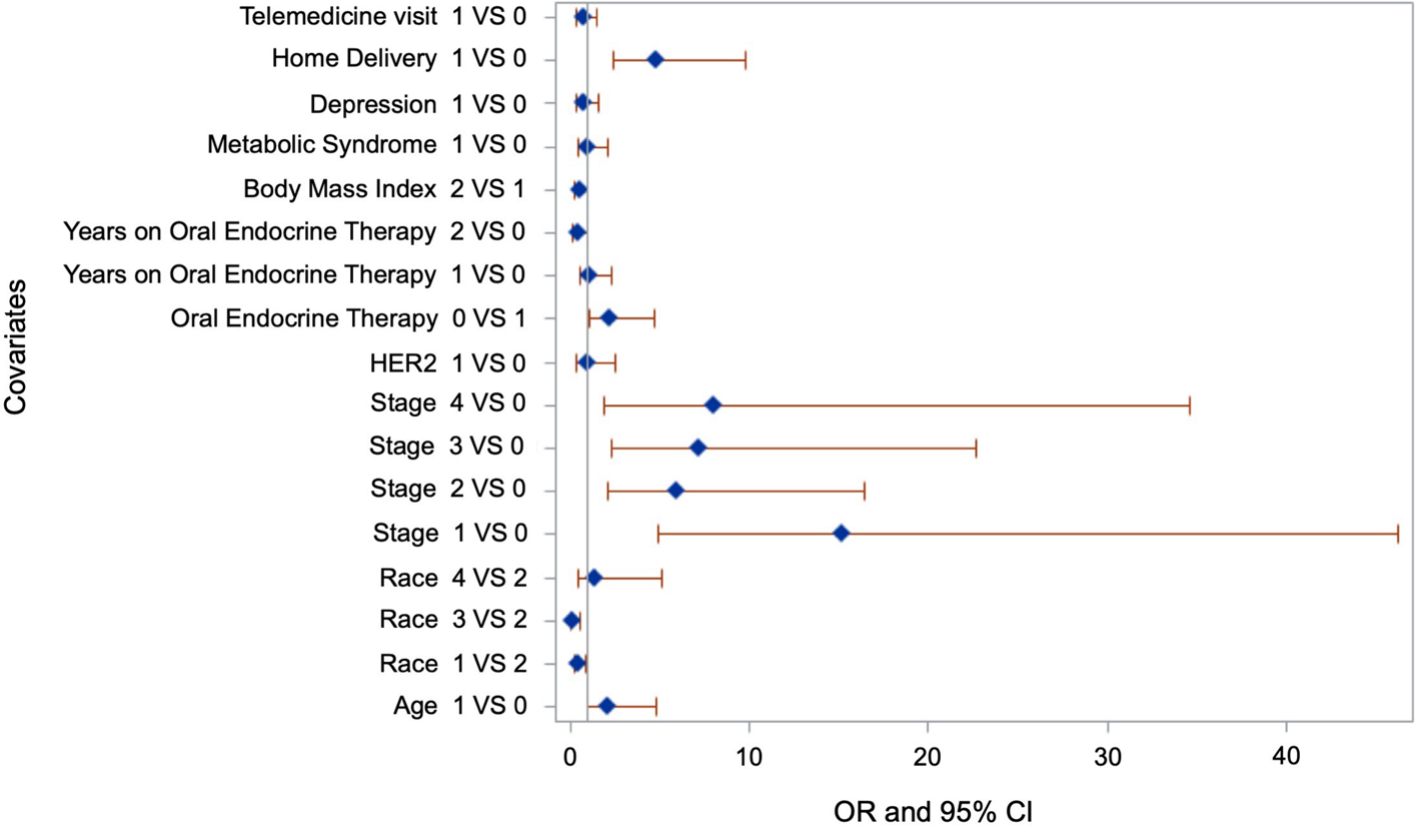
Forest plot for predictors of OET adherence during COVID-19 pandemic. Forest plots were constructed using SAS. The codes for various covariates are as follows: Telemedicine (0 = 0, 1 =  ≥ 1), Home delivery (0 = 0, 1 =  ≥ 1), Depression (0 = No, 1 = Yes), Metabolic syndrome (0 = No, 1 = Yes), Body mass index (0 =  < 30 Ref, 1 =  ≥ 30), Years on Oral Endocrine Therapy (0 =  ≤ 1 year, 1 = 2–3 years, 2 =  ≥ 4 years), Oral endocrine therapy (0 = Tamoxifen, 1 = Aromatase inhibitor), HER2 (0 = HER2-negative, 1 = HER2-positive), Stage (0 = stage 0 or Ductal/lobular hyperplasia, 1 = stage I, 2 = stage II, 3 = stage III, 4 = stage IV), Race (1 = Black/African American, 2 = Hispanic/Latino, 3 = White/Caucasian, 4 = Others), Age (0 = Below 65 years, 1 = 65 years and above)

Patients who were diagnosed with invasive BC (stages 1–3) were more likely to be adherent compared to those diagnosed with non-invasive tumors or ductal/lobular hyperplasia. Patients using home delivery at least once were more likely to be adherent compared to those not using home delivery (OR, 25.42; 95% CI 7.44–86.81) (Table 3). The proportion of patients using home delivery was not significantly different between racial/ethnic groups. The forest plot showing the odds-ratio during COVID-19 pandemic is presented as Fig. 2.

**Table 3:** Demographic and clinical characteristics associated with nonadherence to OET during the COVID-19 pandemic

Variable	Adjusted OR	95% CI	*P*-value
*Age*			
Below 65 years	Ref		
65 years and above	0.77	0.34–1.70	0.51
*Race/Ethnicity*			
Hispanic/Latino	Ref		
Black/African American	1.10	0.51–2.34	0.80
White/Caucasian	0.45	0.11–1.85	0.27
Others	3.08	0.82–11.51	0.09
*Body Mass Index*			
< 30	Ref		
≥ 30	0.72	0.37–1.41	0.34
*Stage*			
0 or Ductal/lobular hyperplasia	Ref		
I	3.95	1.27–12.30	0.01^a^
II	3.54	1.15–10.90	0.02^a^
III	4.82	1.38–16.84	0.01^a^
IV	3.13	0.68–14.35	0.14
*HER2*			
HER2-negative	Ref		
HER2-positive	2.26	0.50–10.27	0.43
*Oral endocrine therapy*			
Tamoxifen	Ref		
Aromatase Inhibitors	1.14	0.54–2.39	0.72
*Years on oral endocrine therapy*			
≤ 1 year	Ref		
2–3 years	0.76	0.37–1.56	0.46
≥ 4 years	0.59	0.24–1.49	0.26
*Metabolic syndrome*			
No	Ref		
Yes	0.82	0.39–1.72	0.61
*Depression*			
No	Ref		
Yes	1.20	0.55–2.64	0.63
*Home Delivery (during COVID-19 pandemic)*			
0	Ref		
≥ 1 home delivery	25.42	7.44–86.81	< 0.0001^a^
*Telemedicine (during COVID-19 pandemic)*			
0	Ref		
≥ 1	1.5	0.71–3.21	0.27

Abbreviations: CI- Confidence interval; OET = Oral endocrine therapy; OR = Odds ratio

A* p*-value ≤ 0.05 was considered statistically significant

^a^Statistically significant

## Discussion

### Statement of key findings

This was a retrospective, single-center study evaluating the impact of the COVID-19 pandemic on OET adherence in racial/ethnic minority patients with low SES. We report that OET adherence was low and was further reduced during the pandemic in patients seen at the Harris Health System that serves the indigent population of Houston. At Harris Health, 45.9% of patients are uninsured, 21.2% are on Medicaid or Children's Health Insurance Program, 12% are on Medicare, and 19.9% are on commercial or other funding. Even before the pandemic, OET adherence was low at only 57% in patients with low SES. This was lower than the OET adherence of 82% at the Houston Methodist Hospital serving insured patients in the same geographic location of Houston before the pandemic in our previous study [42]. The pandemic further and significantly reduced OET adherence to only 44% of Harris Health patients. COVID-19 has been found to negatively impact cancer outcomes in racial/ethnic minorities and patients with low SES [43–45]. While access to life-saving medications such as OET was provided to Harris Health patients by introducing home delivery, it was not utilized by all patients, which significantly impacted adherence during the pandemic. Identifying prohibitive factors for using home delivery is necessary to optimize outcomes in this patient population.

### Strengths and weaknesses

Strengths of our study include the significance of the research, novelty of our findings, and the study design. Harris Health System currently does not have an existing program to improve adherence. While our finding of high OET nonadherence in racial/ethnic minorities with low SES is consistent with published reports, we have several novel findings as well. For example, the impact of COVID-19 on OET adherence, although expected, has not been reported before. Also, our study has identified a major area of unmet clinical need, in the setting of improving OET adherence for BC prevention, which is a largely ignored area of research until this point. Our study highlights home delivery as a simple solution to improve OET adherence that can also be applicable to other chronic medications and relevant in future pandemic or natural disaster settings. The findings of this study can also be used to design and implement future interventions and programs customized to African American and Hispanic patients to improve their long-term adherence. Our study provides critical information for future studies conducting a real-time assessment of adherence and implementation of interventions in the ambulatory oncology clinic.

Some limitations of this study are described below. This was a retrospective study conducted in one center, which can limit the generalizability of findings to similar centers and geographic locations. This is a hypothesis generating exploratory analysis. Given the scarcity of studies specifically conducted in racial/ethnic minority patients with low SES, future more theory-driven studies that take the findings of this study into consideration are warranted. The relatively small sample size may have impacted the power to detect significant differences and lead to wide confidence intervals with some variables. Therefore, additional larger studies are needed to validate our findings and confirm the demographic and clinical characteristics associated with non-adherence in racial/ethnic minorities with low SES. Refilling the medications does not guarantee the patient actually consumes the medication as prescribed. Additionally, residual uncontrolled confounding may exist as some variables that can impact adherence were not controlled for, like educational level, income, family support, and other comorbidities. Despite these limitations, the study adds to the growing literature on low OET adherence rate among racial/ethnic minorities with low SES, underscoring the need for tailored interventions to enhance adherence in this population.

### Interpretation

We found that stage was associated with OET nonadherence in our population. Patients at higher risk of invasive BC, namely those diagnosed with non-invasive BC or with ductal/lobular hyperplasia, were less likely to be adherent to OET.

Most studies to date have evaluated adjuvant OET adherence in the setting of early-stage BC treatment, not for prevention [20–25]. Our study emphasizes an important need to evaluate barriers to OET adherence in the prevention setting and for tailored interventions to improve OET adherence since it reduces the risk of new BC diagnosis.

Our results of lower OET adherence in Black/African Americans are consistent with published literature [21–25]. African Americans have a higher incidence of certain cancers and face greater hurdles to cancer prevention, detection, and treatment [46]. As a result, African Americans have the highest death rate and shortest survival of any racial/ethnic group for most cancers [46–48]. Despite slightly lower rates of breast cancer (BC) diagnosis, African American women are over 40% more likely to die from it than Caucasians in Texas and the U.S. [27, 28, 46, 47, 49]. The most common barrier to OET adherence reported by patients and physicians is adverse drug reactions [50–52]. African American women more often report postmenopausal symptoms and joint pain with OET than Caucasians [53, 54]. They also more often believe that their recurrence risk would not change if they stopped OET and report forgetting to take it regularly [53–56]. Therefore, patient-centered interventions addressing these key barriers to OET adherence in African American women, especially those with low SES, are urgently needed.

A large subset (62%) of patients in our study was obese or extremely obese, which was also associated with nonadherence. Higher body weight has been associated with worse BC-specific outcomes in patients, and body weight management is linked to favorable BC-specific survival [17, 57–59]. It is possible that patients who are more health-conscious in general are more likely to maintain their ideal body weight and stay on therapy. Therefore, adequate patient education as well as interventions to improve overall health including weight management and OET adherence are necessary to improve overall BC outcomes in HR + BC patients with low SES.

### Further research

Our findings were communicated to the breast medical oncology and the cancer prevention team as well as the Pharmacy Department at the Harris Health Hospital. Department of Pharmacy had instituted the home delivery option during the pandemic. Since not using home delivery adversely affected OET adherence, encouraging patients to use it could help improve their OET adherence. The home delivery option is still available to all Harris Health patients, and its optimized use could lower the burden of the pandemic on patients with low SES. Future directions for research include developing patient-centered interventions and testing their feasibility and effectiveness to improve treatment outcomes and survival in racial and ethnic minorities with lower SES.

## Conclusion

In conclusion, OET adherence is a major concern in racial/ethnic minorities with low SES with HR + BC. The COVID-19 pandemic adversely affected OET adherence. Therefore, patient-centered interventions tailored to Black/African American patients and those using OET for prevention are urgently needed to improve BC outcomes in this population.
